# Toward Standardized Assessment of Dynamic Subjective Visual Vertical: Effects of Visual Stimulus Intensity in Health and Multiple Sclerosis

**DOI:** 10.3390/medicina61081482

**Published:** 2025-08-18

**Authors:** Tautvydas Klėgėris, Diego Kaski, Renata Balnytė, Virgilijus Uloza, Alina Kuzminienė, Ingrida Ulozienė

**Affiliations:** 1 Department of Otorhinolaryngology, Faculty of Medicine, Lithuanian University of Health Sciences, 44307 Kaunas, Lithuania; virgilijus.ulozas@lsmu.lt (V.U.); alina.kuzminiene@lsmu.lt (A.K.); ingrida.uloziene@lsmu.lt (I.U.); 2 Department of Clinical and Movement Neuroscience, University College London, London WC1E 6BT, UK; d.kaski@ucl.ac.uk; 3 Department of Neurology, Medical Academy, Lithuanian University of Health Sciences, 44307 Kaunas, Lithuania; renata.balnyte@lsmu.lt

**Keywords:** multiple sclerosis, balance, subjective visual vertical, dynamic visual stimulus, gravity perception, visual dependence

## Abstract

*Background and Objectives*: Decreased balance function in multiple sclerosis (MS) patients is influenced by impaired gravity perception, which can be measured by the subjective visual vertical (SVV) test. The value of this test can be increased by executing it in a moving visual background (i.e., dynamic SVV). However, clinicians and researchers use varying dynamic stimulus properties due to the lack of consensus on optimal parameters for reliably distinguishing between health and disease. *Materials and Methods*: To evaluate how dynamic visual stimulus intensity affects the perception of verticality in patients with MS and healthy individuals. *Materials and Methods*: We assessed static and dynamic SVV in 31 MS patients with dizziness and 32 age- and sex-matched controls using the virtual reality application VIRVEST. We evaluated the effects of modifying two parameters in dynamic SVV testing: rotation velocity (10°/s, 30°/s, and 60°/s) and visual field coverage (small vs. large). *Results*: The median of static SVV deviations was significantly greater in the MS group (1.8° vs. 0.9°). The mildest dynamic stimulus intensity of 10°/s, with a small visual field coverage, yielded the greatest discriminatory capacity to differentiate between the groups (AUC = 0.897; *p* < 0.001). This stimulus elicited a median SVV deviation of 4.3° in the MS group and 2.1° in the control group (*p* < 0.001) while also inducing significantly lower test-induced dizziness compared with stronger stimuli. Median visual dependence values measured at 10°/s with a small visual field coverage were 4.2 in the MS group and 2.02 in the control group (*p* < 0.001), also yielding the greatest AUC values compared to stronger stimuli (AUC = 0.828; *p* < 0.001). *Conclusions*: Our results support the use of relatively mild dynamic stimulus intensity. Future studies are encouraged to evaluate different dynamic stimulus parameters and patient populations.

## 1. Introduction

Gravity is a constant and fundamental force around which all human motor behaviour and balance control must be organized. Thus, a precise internal representation of the gravitational vector (i.e., verticality) is essential for maintaining postural stability. It is a demanding activity for the central nervous system (CNS), requiring the integration of multiple sensory inputs, each weighted according to its reliability and combined with prior knowledge [1]. Several studies have reported that impaired gravity perception may be involved in the pathophysiology of dizziness and decreased balance function in individuals with multiple sclerosis (MS) [2,3], the most common neurodegenerative disease in young adults [4]. Balance disorders are highly prevalent in people with MS, affecting approximately 50–80% of patients during the course of the disease [5]. It is a major contributor to disease-related disability, adversely affecting the quality of life in this patient population [6]. Therefore, quantifying such perceptual disturbances might contribute to a more accurate assessment of MS patients‘ functional status and potentially facilitate tailored rehabilitation planning.

The subjective visual vertical (SVV) test is a reliable method of evaluating verticality perception [7] and detecting central vestibular deficits in patients with MS [8,9]. Brandt and Dieterich introduced the SVV as a sensitive tool for diagnosing vestibular tone imbalance, especially in central otolith disorders [10]. Later, the ”bucket method”—a cost-effective bedside SVV test was validated in 2009 [11]. More recently, virtual reality (VR)-based SVV assessments have been validated in both research and clinical settings [12,13].

Unlike most conventional vestibular tests, SVV relies on central processing of sensory inputs related to spatial orientation rather than on vestibular reflexes. This makes it especially useful for identifying subtle impairments in spatial orientation that may be overlooked by reflex-based tests, which tend not to probe perceptual dysfunction present in many neurological disorders. Its high sensitivity in acute vestibular disorders, good test-retest reliability, and capacity to quantify vestibular perception make SVV a valuable component of the otoneurological assessment battery [14,15]. Implementing a moving background visual stimuli further challenges the recognition of true gravitational vertical [16], this being significantly more pronounced in individuals with peripheral or central vestibular dysfunction than in healthy controls [13,17]. Such dynamic SVV is most easily achieved using virtual reality (VR)-based systems. The exact mechanism underlying vestibular patients’ susceptibility to visual motion sensitivity is unknown; however, it is thought to involve a sensory reweighting process in which greater importance is given to visual inputs, relative to vestibular and somatosensory inputs, for spatial orientation [18]. This overreliance on visual information, which is also observed in patients with MS [9], is known as visual dependence (VD).

Dynamic SVV demonstrates higher sensitivity in chronic and subacute vestibular disorders (compared to static SVV) [19], is effective for monitoring central compensation following an acute vestibular insult [20], and is frequently used to quantify the degree of VD [21]. However, the lack of standardization in the test‘s methodology leads to substantial variability in results and limits comparability across studies. Different intensities of dynamic stimuli have been employed, with rotational velocities of background motion ranging from 4 to 120 degrees per second. As a result, findings on verticality misperception in dynamic conditions vary widely, from less than 1° to over 10°, even in similar patient populations and between healthy controls [12,13,17,22,23]. It remains unclear whether the intensity of the dynamic stimulus affects diagnostic yield and patient comfort during testing in neurologic patient populations. Therefore, this cross-sectional observational study aims to explore the influence of visual stimulus intensity on dynamic SVV performance, VD, and test-induced dizziness in MS patients with balance disturbance. Primary endpoints include the magnitude of SVV deviations, VD scores, and subjective dizziness scores across different stimulus conditions. In addition, we evaluated the diagnostic ability of dynamic SVV at each stimulus intensity to distinguish MS patients from healthy controls. We assessed the effects of modifying two parameters in dynamic SVV testing: rotation velocity and visual field coverage.

## 2. Methods

The study was conducted at the Departments of Otorhinolaryngology and Neurology, Lithuanian University of Health Sciences Hospital, Kaunas, Lithuania. The research protocol was reviewed and approved by the Kaunas Regional Ethics Committee for Biomedical Research (Approval No. BE-2-18; date of approval: 30 January 2024). The study adhered to the institution’s ethical guidelines and the principles of the Declaration of Helsinki. All participants were informed about the study protocol and provided written informed consent prior to their participation in the study.

### 2.1. Subjects

Thirty-one adult patients (25 female) diagnosed with relapsing–remitting MS were recruited into this study from the Neurology Department of the Lithuanian University of Health Sciences Hospital. The diagnosis of MS was established according to the globally accepted and revised McDonald criteria [24]. The severity of dizziness was assessed using a validated Lithuanian version of the Dizziness Handicap Inventory (DHI) [25]. The median DHI score of MS patients was 22 (10–78). Peripheral vestibular function was evaluated using caloric testing with videonystagmography (VNG system VN15/VO25, Interacoustics, Middelfart, Denmark), which was normal in all patients. The video head impulse test was not available. MRI data on demyelinating lesion location or activity were not collected for this study, as previous research did not identify its influence on SVV or VD [9]. MS-related disability was evaluated using the Expanded Disability Status Scale (EDSS) [26]. The median EDSS score was 2.5 (1.5–6.0). The exclusion criteria for the MS group included the following:

- Significant MS-related disability that interfered with task comprehension and/or execution;
- Uncorrected ophthalmological impairment that prevented consistent task completion;
- Age greater than 55 years;
- Other neurological, vestibular, or otological disorders.

Thirty-two healthy adult volunteers (24 female) without complaints of dizziness, vertigo, or unsteadiness in the past six months were recruited in the control group. The exclusion criteria for control group participants were as follows:

- Any known neurological or otological diseases;
- Age greater than 55;
- Significant uncorrected ophthalmological impairment.

The groups were matched for age (*p* = 0.669) and sex (*p* = 0.59).

### 2.2. Sample Size Justification

The sample size was determined using the following formula for comparing two independent means:

$$n=\frac{\left(s_{1}^{2}+s_{2}^{2}\right)\cdot {\left(z_{q1}+z_{q2}\right)}^{2}}{\Delta^{2}}$$

where

n = required sample size per group,

$s_{1}^{2}$ = variance of the outcome variable in the first group (patients),

$s_{2}^{2}$ = variance of the outcome variable in the second group (controls),

$z_{q1}$ = Z-score corresponding to the desired statistical power (0.842 for 80% power),

$z_{q2}$ = Z-score corresponding to the chosen significance level for a two-tailed test (1.96 for α = 0.05),

$\Delta^{2}$ = minimum clinically meaningful difference between groups (1°).

Based on variances of 0.59 and 2.02 from previous data [9], a significance level of 0.05, and power of 80%, the required sample size per group was approximately 21.

### 2.3. Experimental Setup

**Figure 1:**
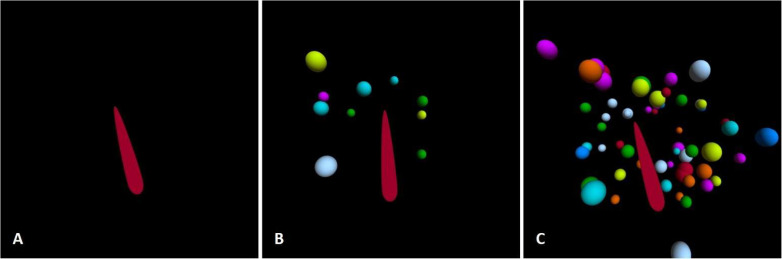
Virtual reality scene for SVV testing: (**A**) static testing; (**B**) dynamic testing with small coverage of the visual field; (**C**) dynamic testing with increased coverage of the visual field.

Subjective visual vertical was assessed using the Oculus Quest 2 virtual reality headset (Meta Platforms, Inc., Menlo Park, CA, USA; CE-marked and FCC ID: 2AGOZMH-A), equipped with the SVV measurement application VIRVEST, which has been previously validated for both healthy individuals and patient populations [9,12]. Subjects were seated upright on a chair in a darkened room to prevent additional visual cues. The VR application displayed a 3D rod positioned in a primary gaze, deviating from the gravitational vertical at random angles between 10° and 15°. Participants were given a wireless joystick and instructed to align the rod with what they perceived as vertical before pressing the confirmation button. When the rod was perceived and confirmed as vertical, the result was automatically saved, and a new trial began. Every participant completed nine tests: static SVV and four dynamic stimulus intensities, each presented in both clockwise (CW) and counter-clockwise (CCW) directions. Each test consisted of 6 trials, resulting in a total of 54 SVV adjustments per participant. During the static tests, the rod was presented against a blank black background without additional visual cues or stimuli (Figure 1A). Dynamic tests consisted of the same rod presented among 3D spheres, with random spatial distribution across the visual field and rotated in the frontal plane in either CW or CCW directions (Figure 1B,C). The initial dynamic stimulus involved ten spheres rotating at a velocity of 10° per second. The intensity of the dynamic stimulus could then be modified by increasing either the rotation velocity or the number of spheres (without changing their size range or velocity).

Each participant began with six trials of the static SVV. The subsequent dynamic tests were then administered in a pseudo-random order, each in blocks of 6 trials:

- 10 spheres, 10°/s.
- 10 spheres, 30°/s.
- 10 spheres, 60°/s.
- Increased visual field coverage (IVFC)—60 spheres, 10°/s (Figure 1C).

The order of dynamic SVV test conditions was varied across participants in a quasi-random manner at the discretion of the examiner. Although no formal randomization procedure was used, efforts were made to avoid systematic order effects. Rotational velocities were selected based on a review of the literature, with 10°/s and 30°/s being the most commonly used, and values below 10°/s or above 60°/s representing the extremes.

Quantitative data was obtained as the angle between gravitational and the subject’s confirmed verticals automatically measured by the application at a precision of 0.1°. The mean SVV deviation angle was calculated from six trials for each test. Rightward deviations were denoted as positive values, and leftward deviations were denoted as negative values. The time intervals between each test were no shorter than 1 min.

After completing the dynamic SVV at each specific intensity, participants were asked to assess their discomfort—described as dizziness, visual vertigo, or general unpleasantness during the testing—and mark it on a visual analogue scale (VAS) ranging from 0 (no dizziness) to 100 (worst imaginable dizziness). The corresponding numerical value was recorded to quantify the severity of discomfort induced by dynamic tests.

### 2.4. Measuring the Visual Dependence

VD reflects the extent to which a disorienting visual stimulus affects the perception of verticality. The most common method for quantifying VD is to calculate the mean difference between the dynamic (CW and CCW) and static SVV adjustments [9,27]. Therefore, in this study, the following formula was used to calculate VD: VD = (|SVV_cw_ − SVV_static_| + |SVV_ccw_ − SVV_static_|)/2.

### 2.5. Statistical Analysis

Quantitative data were tested for normality using the Shapiro–Wilk test, which indicated a non-normal distribution (*p* < 0.05). Therefore, quantitative variables are presented as medians, along with their minimum and maximum values. The non-parametric Mann–Whitney U test was used to compare quantitative data between the MS and control groups. For comparisons of dependent quantitative data (i.e., repeated measures), the Wilcoxon signed-rank test or the Friedman test was applied. Categorical variables were compared using the chi-square test. Spearman‘s correlation was used to assess the relationship between quantitative variables. Receiver operating characteristic (ROC) curves were generated to assess the ability of SVV deviation values at each stimulus intensity to discriminate between MS patients and healthy controls. Sensitivity (true positive rate) was plotted against 1–specificity (false positive rate) for each threshold. The area under the curve (AUC) was calculated to evaluate overall discriminatory performance. Statistical analyses were conducted using IBM SPSS Statistics version 29.0 for Windows. Differences were considered statistically significant if *p* < 0.05.

## 3. Results

Patients in the MS group exhibited significantly higher absolute SVV deviations during static and all dynamic tests compared to controls. VD values were significantly greater in the MS group when tested with dynamic stimuli at 10°/s, 30°/s, and IVFC, but no significant difference was found at 60°/s (Table 1).

SVV deviations were significantly larger in all dynamic compared to static tests across both MS and control groups (*p* < 0.001, Wilcoxon test). To assess the effect of visual stimulus intensity on the perception of verticality, dynamic SVV performance was compared within each group across all intensity levels. In the MS group, a statistically significant difference was observed between the mildest stimulus (10°/s) and each of the stronger stimuli. However, no significant differences were found among the stronger stimuli themselves. In the control group, statistically significant differences were observed between the different visual stimulus intensities in each pair. The results of within-group comparisons for the effect of visual stimulus intensity on dynamic SVV performance are presented in Table 2.

In both groups, VAS scores for visually induced dizziness increased significantly with greater stimulus intensity, irrespective of whether the intensity was modulated through rotation velocity or visual field coverage (*p* < 0.05 for each within-group comparison, Wilcoxon test). Test-induced dizziness was also compared between the groups. MS patients reported significantly higher VAS scores than healthy controls at the 10°/s stimulus intensity. However, no significant differences were observed between the groups at higher stimulus intensities (Table 3).

In the MS group, a significant moderate positive correlation was found between VAS scores and VD at 10°/s and 30°/, but not at 60°/s or 10°/s IFVC stimuli. In the control group, a significant, strong positive correlation was found between VAS scores and VD at 10°/s (*ρ* = 0.701, *p* < 0.001), 30°/s (*ρ* = 0.757, *p* < 0.001), and 60°/s (*ρ* = 0.633, *p* < 0.001), but not at 10°/s IFVC stimuli.

VAS and DHI scores of MS patients moderately correlated at 10°/s, 30°/s, and 10°/s IFVC stimuli, but did not correlate significantly at 60°/s stimulus (*p* = 0.146). We did not identify any significant correlations between DHI scores and either SVV deviations or VD (Table 4).

**Figure 2:**
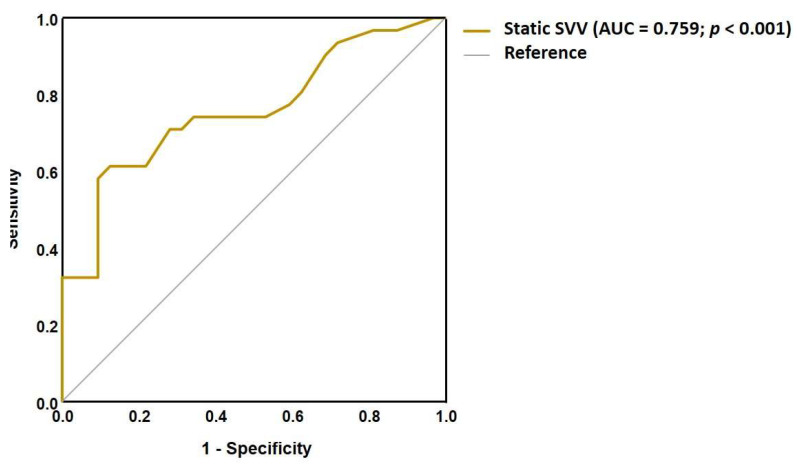
ROC curve for the static SVV test to differentiate MS patients from healthy controls.

**Figure 3:**
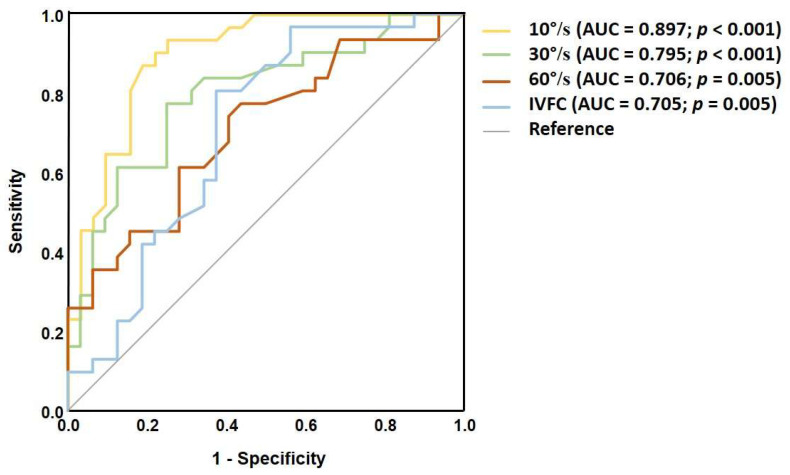
ROC curves for each visual stimulus intensity of the dynamic SVV test to differentiate MS patients from healthy controls.

**Figure 4:**
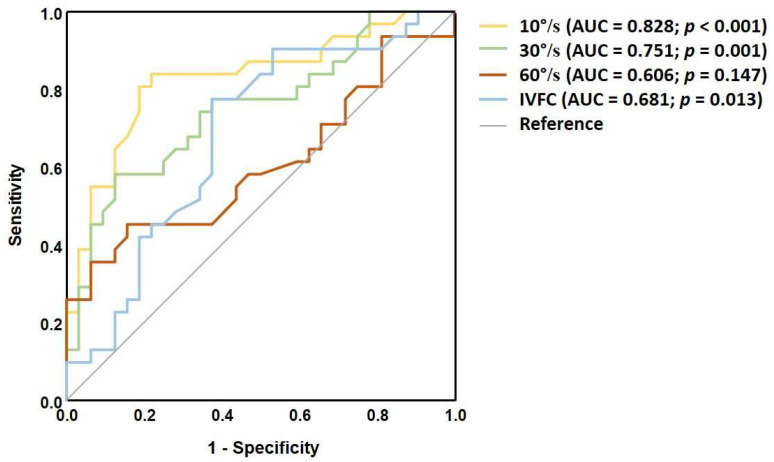
ROC curves for VD values at each visual stimulus intensity to differentiate MS patients from healthy controls.

To evaluate the ability of each SVV test to distinguish dizzy MS patients from healthy controls, a ROC curve analysis was performed. Static SVV demonstrated a fair discriminatory capacity with area under the ROC curve (AUC) of 0.759; *p* < 0.001 (Figure 2). Dynamic SVV at 10°/s yielded the highest AUC compared to stronger stimulus intensities, indicating good discriminatory ability (AUC = 0.897; *p* < 0.001) (Figure 3). Additionally, the VD measurement also achieved its highest AUC at the 10°/s visual stimulus (AUC = 0.828; *p* < 0.001) (Figure 4). The cut-off values for each dynamic stimulus intensity are presented in Table 5.

## 4. Discussion

The present study aimed to investigate how the intensity of dynamic visual stimuli affects verticality judgments, visually induced dizziness, and the discriminatory capacity of the dynamic SVV test in distinguishing dizzy MS patients from healthy controls.

### 4.1. Group Differences in Static SVV Performance

Firstly, in the MS group, we identified greater errors in the perception of visual verticality during conventional static SVV testing. These findings build on previous knowledge of altered gravity estimation in ptients with MS. The exact mechanism by which MS alters the perception of verticality remains unclear. Demyelinating lesions can disrupt widely distributed central vestibular pathways encoding the head-in-space orientation [1]. It may also interfere with the Bayesian integration of sensory information (mainly from vestibular, visual, and proprioceptive inputs) that underlies spatial orientation. Furthermore, accurate spatial orientation depends on the brain’s ability to weigh sensory inputs according to their relative reliability [28]. This dynamic weighting process may also be compromised by MS-related damage to relevant neural structures.

Although statistically significant, the difference in static SVV median deviations between the groups was not substantial, reaching only 0.9 degrees. However, as a result of vertical body posture, humans must operate on a relatively constricted base of support in addition to a heightened center of mass, which makes the maintenance of uprightness vulnerable to deviations from the axis of gravity [7]. Therefore, even subtle impairments in identifying the gravity vector precisely may contribute to worse postural control outcomes. This has been demonstrated in neurological patients with Parkinson‘s disease [29], stroke [30,31], MS [2], as well as in older individuals with balance disorders [21]. Based on these findings, the SVV test can be considered a valuable tool for assessing spatial orientation and potentially guiding targeted rehabilitation.

### 4.2. The Effects of Dynamic Stimulus Intensity

The issue of the SVV test‘s methodological inconsistency has been addressed by Piscicelli and Pérennou, who conducted a systematic review and proposed standardization suggestions for testing the static SVV [32]. However, to our knowledge, no studies have directly aimed at standardizing dynamic SVV, which provides critical data on visual–vestibular cue interaction in the CNS. The present study approached this problem by assessing how variations in visual stimulus intensity, via rotation velocity and the extent of visual field coverage, affect the dynamic SVV test’s performance in distinguishing dizzy MS patients from healthy controls. Our findings suggest that the mildest visual stimulus intensity, consisting of ten spheres rotating at a velocity of 10°/s, may be the most appropriate for detecting subtle spatial orientation impairments and evaluating the extent of visual field dependence in MS patients. Whilst significantly greater SVV deviations were observed during all dynamic tests in the MS group, ROC analysis revealed that the mildest stimulus demonstrated the greatest discriminatory capacity (the highest AUC value). Also, it was the only dynamic test that could be considered as “good” (AUC 0.8–0.9) based on ROC analysis and approached the “excellent” category (AUC ≥ 0.9). This may have several explanations. First of all, a visual rotation of low velocity generates a stronger self-motion illusion (i.e., vection) because of the low-frequency preference of the visual system. This is the basis of the ”moving train“ illusion when our brain is tricked into thinking our train is moving when, in fact, the other train next to ours is pulling out slowly [33]. The vection in this situation would not be produced if the nearby train moved at, for example, 50 km per hour. Likewise, a dynamic SVV test would potentially be useless with very high rotation velocities of 100°/s or higher, as it would be significantly distinct from the velocities at which the human body usually moves in the standard environment (walking, moving our head, etc.). It was shown that individuals with MS tend to be more visually dependent [9], thus their threshold for vection and visually-induced biases in verticality perception should be considerably lower. This is also apparent in the VAS scores of test-induced dizziness, as MS patients reported significantly greater dizziness at 10°/s compared to controls, whereas the differences were insignificant at stronger stimuli. At low velocities, however, healthy individuals with intact spatial orientation may appropriately weigh the sensory cues according to their reliability and utilize more reliable sensory information provided by vestibular and proprioceptive inputs. When the velocity of visual rotation increases up to 30–60°/s, it potentially reaches the threshold for vection in healthy individuals, and between-group differences in SVV errors become slightly less pronounced. This is also supported by the comparison of VD scores between the groups. VD measured at 10°/s dynamic stimulus demonstrated the highest diagnostic yield based on ROC analysis, and did not differ significantly when measured at 60°/s. Correspondingly, we hypothesize that the IVFC test yielded slightly worse diagnostic performance because of the greater self-movement illusion produced in healthy controls. Visually induced SVV errors appear to saturate between 30°/s and 60°/s in patients with MS, since the within-group differences between these stimulus intensities were not statistically significant. A similar pattern was observed in the control group, as the medians of SVV deviations at 30°/s and 60°/s were almost identical (3.7 and 3.8, respectively), although the Wilcoxon repeated measures test revealed statistical significance. Comparable saturation effects were observed in previous studies with different patient populations [34,35]. Therefore, the use of rotation velocities greater than 60°/s is likely unwarranted.

### 4.3. Test-Induced Dizziness

Another important factor to consider when selecting dynamic stimulus intensity is test-induced dizziness. Higher rotation velocities elicited significantly stronger sensations of movement in both groups, as measured by VAS scores. Given that patients who undergo SVV testing are more prone to visual vertigo due to vestibular or neurological conditions, discomfort during dynamic testing may discourage them from continuing or repeating the test.

### 4.4. Clinical Applicability

Although the SVV test is not used for direct diagnosis, it can provide valuable insights into the pathophysiology of vestibular symptoms like postural instability or visually induced dizziness. This is particularly relevant in neurologic patients, such as those with MS, where peripheral vestibular function is often intact, and abnormal perceptual test results may be the only indication of central vestibular involvement. The dynamic SVV test is a relatively simple way to assess visual dependence, which tends to increase as MS progresses [9]. Because of this, it could be a useful tool for monitoring changes in central vestibular function over time, and for guiding rehabilitation strategies focused on symptom management.

Importantly, increased VD should not be considered pathological on its own, as some healthy individuals may naturally exhibit a higher reliance on vision for balance [18]. However, it is significantly more prevalent among subjects with underlying vestibular or neurological impairment [17,18]. The dynamic SVV remains one of the few methods to assess VD; therefore, the consistency in the test‘s methodology is crucial.

### 4.5. Limitations

To our knowledge, this is the first study directly aimed at identifying the most applicable parameters for dynamic SVV. However, it has several limitations. The sample size was calculated using a formula based on assumptions of normal distribution and equal variances, which are associated with parametric tests. However, due to non-normality in the data, mainly the Mann–Whitney U test—a non-parametric alternative—was used for group comparisons. As a result, the sample size estimate may not perfectly reflect the power of the non-parametric test, although it is generally considered a conservative approximation. Moreover, there are additional parameters of the dynamic stimulus that we did not evaluate, such as the use of a central “protected” area, which prevents overlap between the spheres and the rod. Thus, rotation velocity and the extent of visual field coverage may not be the only parameters affecting the performance of the dynamic SVV. Another limitation of the present study is that the order of dynamic SVV conditions was not formally randomized. While the examiner varied the sequence across participants to reduce predictability, the lack of a structured randomization protocol may have introduced order effects or fatigue-related biases. Lastly, testing the static SVV alongside four different dynamic stimulus intensities takes a relatively long time (around 30 min) and may potentially cause fatigue, which can affect test performance. Despite these limitations, our findings support the use of dynamic stimuli with relatively low rotation velocity and visual field coverage.

## 5. Conclusions

This study assessed the effect of varying the visual stimulus intensity on dynamic SVV test performance in patients with MS and healthy controls. Among the tested parameters, a mild dynamic visual stimulus—10 rotating spheres at 10°/s—provided the highest discriminatory accuracy between MS patients and healthy controls, while also minimizing test-induced dizziness. These findings highlight the potential of low-intensity dynamic SVV testing as a sensitive and well-tolerated method for detecting central vestibular dysfunction and increased visual dependence in MS. Standardizing such parameters could improve comparability across studies, facilitate interpretation of results, and support the development of more consistent diagnostic protocols. Future studies are encouraged to evaluate different dynamic stimulus parameters and patient populations.

## Figures and Tables

**Table 1:** Median (min.–max.) values of absolute static and dynamic SVV deviations, and VD in both groups.

	MS Group	Control Group	*p*-Value *
Static SVV, median (min.–max.)	1.8 (0.1–4.6)	0.9 (0–2.4)	<0.001
Dynamic SVV deviations, median (min.–max.)			
10°/s	4.3 (2.2–22.7)	2.1 (0.3–7.4)	<0.001
30°/s	6.2 (2.4–23.1)	3.7 (0.5–12.4)	<0.001
60°/s	5.6 (1.4–36.8)	3.8 (1.3–10.4)	0.005
IVFC	6.4 (3.3–20.4)	5.2 (1.3–10.7)	0.005
Visual dependence, median (min.–max.)			
10°/s	4.2 (0.75–22.7)	2.02 (0.15–7.35)	<0.001
30°/s	6.25 (1.65–23.1)	3.65 (0.65–12.4)	<0.001
60°/s	4.2 (0.6–36.85)	3.82 (1.3–10.4)	0.151
IVFC	6.4 (2.8–20.4)	5.15 (0.8–10.65)	0.014

* The Mann–Whitney U test was used. SVV: subjective visual vertical; IVFC: Increased visual field coverage.

**Table 2:** Effect of visual stimulus intensity on dynamic SVV deviations: within-group comparisons.

Pair of Dynamic Stimuli	MS Group (*p*-Value *)	Control Group (*p*-Value *)
10°/s vs. 30°/s	0.006	<0.001
10°/s vs. 60°/s	0.036	<0.001
10°/s vs. IVFC	<0.001	<0.001
30°/s vs. 60°/s	0.563	0.024

* The Wilcoxon test was used.

**Table 3:** Median (min.–max.) VAS values of visually-induced dizziness at each dynamic stimulus intensity are presented and compared between the groups.

Visual Stimulus Intensity	MS Group	Control Group	*p*-Value *
10°/s	20 (0–61)	10 (0–43)	0.018
30°/s	33 (4–80)	25 (0–72)	0.216
60°/s	50 (4–91)	42.5 (0–75)	0.302
10°/s, IVFC	33 (3–72)	34 (5–75)	0.815

* The Mann–Whitney U test was used.

**Table 4:** Spearman correlations between variables at different stimulus conditions in the MS group.

Stimulus	VD vs. VAS	VAS vs. DHI	DHI vs. VD/SVV
10°/s	*ρ* = 0.542 (*p* = 0.002)	*ρ* = 0.523 (*p* = 0.003)	n/s
30°/s	*ρ* = 0.448 (*p* = 0.012)	*ρ* = 0.468 (*p* = 0.008)	n/s
60°/s	n/s	n/s	n/s
10°/s, IVFC	n/s	*ρ* = 0.585 (*p* < 0.001)	n/s

n/s: not significant. VD: visual dependence VAS: visual analogue scale; DHI: Dizziness Handicap Inventory.

**Table 5:** Cut-off values for mean (clockwise and counterclockwise) dynamic SVV deviations at each stimulus intensity and percentage of abnormal cases in the MS group.

Visual Stimulus Intensity	Cut-Off Value(deg)	Abnormal in the MS Group, *n* (%)
10°/s	2.9	29 (93%)
30°/s	3.8	26 (84%)
60°/s	3.9	23 (74%)
10°/s, IVFC	5.5	25 (80%)

## Data Availability

The original contributions presented in this study are included in the article. Further inquiries can be directed to the corresponding author.
